# Extended loneliness. When hyperconnectivity makes us feel alone

**DOI:** 10.1007/s10676-022-09669-4

**Published:** 2022-11-09

**Authors:** Laura Candiotto

**Affiliations:** 1 Institute of Philosophy, Freie Universität Berlin, Habelschwerdter Allee 45, 14195 Berlin, Germany; 2 Centre for Ethics, University of Pardubice, Studentská 95, 532 10 Pardubice II, Czech Republic

**Keywords:** Loneliness, Internet, Online, Extended mind, Background existential feeling, Sherry Turkle

## Abstract

In this paper, I analyse a specific kind of loneliness that can be experienced in the networked life, namely “extended loneliness”. I claim that loneliness—conceived of as stemming from a lack of satisfying relationships to others—can arise from an abundance of connections in the online sphere. Extended loneliness, in these cases, does not result from a lack of connections to other people. On the contrary, it consists in the complex affective experience of both lacking and longing for meaningful relationships while being connected to many people online. The recursive interaction with a digital assistant in a smart flat is my key example for defining the contours of this specific kind of loneliness that emerges when hyperconnectivity becomes pervasive in the user’s daily-life. Drawing on Sherry Turkle’s work and employing the conceptual framework of the extended mind, I analyse the specific characteristics of extended loneliness and explore its phenomenology.

## Introduction

In a recent interview with *The Guardian*, sociologist Sherry Turkle declared that the pandemic “has made us so dependent on forging relationships and maintaining relationships on screen” that “you start out saying the Internet is better than nothing, then suddenly you start saying maybe it is better than everything.”^1^ Turkle is one of the first academics who studied the effects of digital technology and robotics on human psychology. After the enthusiasm of the eighties and the nineties, when as a young professor at MIT she had first-hand experience of the incredible developments of artificial intelligence, robotics, and the Internet, Turkle adopted a more critical position presented in her acclaimed book “*Alone Together*” (2011). In this book, Turkle argued that by increasingly engaging with artificial intelligences and interacting via the Internet, we became prepared to have so-called “relationships with less”. These are relationships that lack essential aspects of human interactions. For instance, when the simulation of emotions replaces actual feelings in the case of interactions with a social robot, 2 or when a “friendship” without commitments last no longer than a few clicks in connecting through social media.

Drawing on Turkle’s work, I will shed light on a specific kind of loneliness that can only be experienced in the networked life. I call it “extended loneliness”. It is extended because it is constituted by the hyperconnectivity of cyberworlds, where technological devices such as smartphones, laptops, and digital assistants as well as platforms, online games, and social media, can be vehicles of loneliness. They become vehicles of loneliness when hyperconnectivity takes the shape of a lifestyle and technological devices are pervasive in the user’s living space. The recursive interaction with a digital assistant in a smart flat is my key example for defining the contours of this specific kind of loneliness that emerges when hyperconnectivity becomes pervasive in the user’s daily-life.

I claim that extended loneliness has specific characteristics particular to the loneliness that can be experienced online. However, I want to avoid a certain tendency in the literature to overemphasise the differences between online and offline relationships.^3^ Not only because nowadays, our lives are a more or less seamless blend of offline and online interactions – quite often with significant overlaps – but mostly because such a clear-cut distinction can make us blind to the more crucial problem concerning loneliness. This, I argue, is the need for affective relationships and the inability to find them fulfilling, both offline and online. A cause concerning online loneliness could be that the empathy experienced online is mostly fictional and deprived of primary intersubjectivity, as it has been argued by Fuchs (2014). I want to add that, crucially, hyperconnectivity amplifies loneliness because it draws us into constant engagement with relationships that are inherently unfulfilling. Hyperconnectivity augments loneliness because constant unfulfillment of relational needs pervades the user’s daily-life and, in the transition to smart living, enters into its private space.

This is not a normative or absolute claim. I do not want to say that extended loneliness is our networked life’s essential and fundamental feeling. Some people can also experience meaningful relationships in online we-spaces, for instance sharing interests with new friends or sustaining a sense of togetherness with people they already know (see Osler 2020, 2021). In this regard, empirical studies show that people mainly use the Internet for keeping in contact with friends and family (Rainie & Wellman, 2012). Online communities can support people in difficult moments of their life, as in coping with grief, as reported by Krueger and Osler (2019). Empirical studies also show that people active on social media not only learn to develop networking skills and strategies for maintaining ties (Rainie & Wellman, 2012), but also tend to be more socially active outside the Internet and take part in larger social networks (Hampton et al., 2009). I do not contradict these findings, but I want to shed light on extended loneliness as a worrying phenomenon that becomes more prevalent the more we move toward smart living and replace offline with online interactions, as it recently happened during the Covid-19 pandemic. Think, for instance, of the struggles of an isolated person during a lockdown trying to fulfil the need for belonging (e.g. fighting the feeling of loneliness) by means of digital technology.

In this paper, I will claim that loneliness – conceived of as stemming from an unsatisfied need for connection – can in fact also arise from an abundance of connections. This new type of loneliness is experienced in the user’s extension through technological devices. Therefore, I suggest that, if we take into account how the Internet changes our relationships, both online and offline, then we have to expand our conception of loneliness to account for extended loneliness, an irreducibly networked kind of loneliness. Extended loneliness does not stem from an actual lack of connections, but it is the complex affective experience of lacking and longing for meaningful relationships while being connected to many people online.

In Sect. 2, I frame my investigation within the extended mind debate and take Jan Slaby’s (2014) phenomenal coupling as my starting point. Then, in Sect. 3, I describe the key features of extended loneliness by the exploration of a daily-life scenario in which the immersion into an online atmosphere and the pervasiveness of online interactions in a private space as a smart flat are crucial. By discussing why this kind of loneliness is extended, I focus in particular on longing for connections in Sect. 4. Finally, by analysing its phenomenology, I present extended loneliness as a background existential feeling in Sect. 5.

## The tethered self and the feeling of extension

In their pioneer paper *The Extended Mind*, Andy Clark and David Chalmers (1998) put forward three notions that received a lot of attention and critical investigation:^4^ extended cognition, extended mind, and extended self. The core idea is that technological devices, from simple tools to sophisticated smartphones, can be integral parts of cognitive processes. By means of these devices, cognitive processes are said to reach beyond the agent’s skull and into the environment. The devices are not mere tools, but proper vehicles of cognition as they externalise mental processes and contents. In doing so, they enhance the agent’s abilities. For example, agents might acquire the ability to remember things without recurring to biological memory through coupling with memory devices used as non-neural data storages.^5^

Clark and Chalmers do not fully develop the idea of an extended self in their initial paper, but put forth the following hypothesis:^6^

What, finally, of the self? Does the extended mind imply an extended self? It seems so. Most of us already accept that the self outstrips the boundaries of consciousness; my dispositional beliefs, for example, constitute in some deep sense part of who I am. If so, then these boundaries may also fall beyond the skin. The information in Otto’s notebook, for example, is a central part of his identity as a cognitive agent. What this comes to is that Otto himself is best regarded as an extended system, a coupling of biological organism and external resources. To consistently resist this conclusion, we would have to shrink the self into a mere bundle of occurrent states, severely threatening its deep psychological continuity. (Clark & Chalmers, 1998, p. 18)

The argumentative line goes as follows: if dispositional states are mental states, and extra-organismic structures can realise dispositional states, then mental states can be realised by extra-organismic structures. Thus, mental states can be extended beyond the skull. In the same vein, if dispositional states are to be considered part of the self, then the self can be partially realised by extra-organismic structures. Finally, the self can be – in part – extended.

Clark further advanced the notion of an extended self in several writings and proposed the idea of a “soft self” (Clark, 2004, 2007). According to this idea “our best tools and technologies literally become us: the human self emerges as a soft self, a constantly negotiable collection of resources easily able to straddle and criss-cross the boundaries between biology and artefact.” (Clark, 2007, p. 278). This is also intertwined with his idea that we are all natural-born cyborgs (Clark, 2004), that is beings who exploit external resources to make their tasks easier, solve them faster, or save energy in doing so. The Internet of things (IoT) has “just” boosted this ability that belonged to the human species from the beginning, Clark claims.

This characterisation of the extended self helps in understanding what Turkle meant when she wrote that the new self of the networked life is best described as a “tethered self” (Turkle, 2011). Turkle has not provided a conceptual analysis of the notion, but has mostly depicted the experience of the tethered self. In particular, she focused on children and teenagers to illustrate what it means to grow up “tethered” (Turkle, 2011: 171–187). She has argued that human beings approach a new state of the self by being tethered to the network through mobile devices (Turkle, 2011: 154–155). She describes the experience as being permeated by a blurring of the confines between physical and virtual. She also pointed to the changes and transformations that this new state of self brings about. For example, the creation of more time through multitasking, the new meaning of privacy in social places by speaking loudly on mobile phones, and the new experience of place when you can “bring” your family and friends with you, wherever you go, through social media.

Analysing the experience of being tethered against the background of Clark’s extended mind and extended self hypothesis helps us to clarify what the conditions for being a tethered self are. A tethered self relies on devices for fulfilling certain cognitive tasks. Devices are not conceived as tools but are integrated into an extended cognitive system. This requires their automatic endorsement. This means that a device is not consciously attended to as a tool, but it is integrated into the extended cognitive system by recursive interactions and automatic endorsements which make it “phenomenologically transparent” to the agent. For Evan Thompson and Mog Stapleton (2009), this means that the device is no longer experienced as an object and the world is experienced through it. Since the technological tool is not attended anymore as an external device in the case of extended cognition, the technological device becomes part of the self. The technological device’s transparency is then a critical condition for the emergence of the tethered self as a self whose experience is mediated by technological devices.

This experience is permeated by certain feelings. Among them, loneliness, as I claim. Many different lines of research have been developed from Clark and Chalmer’s ground-breaking extended approach to cognition, the mind, and the self. The most interesting one in the context of extended loneliness is precisely the extended emotions research programme.^7^ Carter et al., (2016) developed a purely functionalist account of extended emotions on which they are seen as supervenient on extended mental states. Drawing on the cognitivist account of emotions, they claim that emotions can be extended through their nonconscious cognitive appraisal component. For my analysis of extended loneliness, Jan Slaby’s (2014) approach that focuses on the feeling of extension is most helpful because it helps depict what it feels like to be a tethered self. Also, Slaby’s approach (Slaby, 2014) points to extension as immersion in an emotional atmosphere. This helps to better understand the conceptual relationship between hyperconnectivity and the technology’s pervasiveness in the user’s living space, which is crucial for the emergence of extended loneliness in my account.

According to Slaby (2014), the extended emotions programme should not debate about the mental states that are the possible vehicles of emotion extension. Instead, he deems it is more productive to focus on the “phenomenal coupling” of emotions instead, namely the feeling of being one with things and people in one’s surroundings by being immersed in emotional atmospheres or climates. For Slaby, this feeling of extension emerges in the continuous interaction with some expressive environmental structure (Slaby, 2014: 37).^8^ This structure is best conceptualised as “affective scaffolding”, namely environmental resources that contribute to affective regulation (Colombetti & Krueger, 2015), and socio-material structures of affectivity (von Maur 2021). Joel Kruger and Lucy Osler have employed the affective scaffoldings framework to better understand online interactions (Krueger & Osler 2019, Osler 2020, Osler, 2021). Among many other chief concepts, such as online we-space and the echo chamber effect, they suggest understanding the engagement in online worlds as Internet-enabled emotion regulation. They claim that using the Internet as affective scaffolding enables agents to build their techno-social niches and shape their emotional life.

I suggest that the notion of “phenomenal coupling” brings something new to this debate that originated in the works of Krueger and Osler. Slaby’s point is that a feeling of coupling emerges by actively engaging with some affective scaffoldings.^9^ Cognitive coupling, which in Clark and Chalmers is what enables the integration of an external tool into the cognitive system, should be understood in its feeling dimension, as the feeling of fusion with technological devices. Extended loneliness as a feeling of extension is then a particular way of being one with technology. So, I inherit from Clark’s account the understanding of a tethered self as a self whose experience of loneliness is mediated by technological devices. The word “extended” in “extended loneliness” is then technical, i.e., a kind of loneliness vehiculated by technological devices become transparent. But this is not enough. For better appreciating what sort of experience is the one of the tethered self, it is necessary to look at the feeling of being one with technology. For doing so, I suggested approaching the extended emotions debate through Slaby’s approach on phenomenal coupling (Slaby, 2014) and applying it to the immersion into online spaces. What I need to do now is to explain how this specific phenomenal extension is realised. The key concept in this regard is longing. Before I get to integrating longing into my account of extended loneliness, I explore an example that clarifies the focus of my analysis. By means of this example, I further introduce key concepts from the philosophical debate on loneliness (Sect. 3). Then, in Sect. 4 I will come back to longing to explain how loneliness is extended in the online lifestyle.

## The case of extended loneliness

Imagine Stella. Stella is a young woman who lives alone in a so-called smart flat. Returning to her flat after work, Stella interacts with her Internet-enabled digital assistant Cyra for various purposes, such as regulating her flat’s heating, getting entertainment, and writing to-do lists. When reading the brief description of Stella’s interaction with Cyra by Osler and Kruger (2019), one might sense loneliness. One might wonder whether Stella is suffering from loneliness even though – or maybe because – she is constantly interacting with Cyra. Did Cyra not kindly welcome Stella when she opened the door to her flat? Or, did it not (or, should I maybe say “she”?) take care of streaming Stella’s favourite songs while she arrived at home, and did she not also remind her of important tasks, such as calling her mother? Cyra indeed gave Stella a lot of attention. And indeed, receiving attention from others stops one from feeling lonely most of the time. Still, I think that Stella’s life is permeated by loneliness. I am not concerned with the quite obvious way in which Stella is alone by living on her own, with her friends and family members living far away.^10^ I focus on the loneliness expressed in her constant need for help, support, attention, and care from Internet-enabled interactions with Cyra.^11^

Stella and Cyra’s case is different from other forms of technological interactions such as speaking to friends or family members in video chats. In these cases, the exchange is *mediated by* technology, but it is not a direct interaction *with* technology. So, it might be argued that only in the case of interaction *with*, not *through* technology, loneliness can be experienced. However, I don’t think this is the right conclusion. Through the interaction with Cyra, Stella can be connected to thousands or even millions of people. Just think about some of Cyra’s quite simple functions such as reading Stella’s social media news feed out loud, or streaming songs from a shared-queue social listening app. But letting Cyra execute all these tasks is not the same as doing them on a smartphone. The reason is that Cyra’s functionality is integrated into Stella’s entire private environment. Through Cyra, Stella’s online interactions physically surround her and create a techno-social niche in which Stella is always online.^12^ This is precisely what Slaby (2014) meant by the feeling of extension as the experience of immersion into an expressive environmental structure, which I presented in the previous section. So, my target cases are about ‘smart’ living. The case of the interaction with a digital assistant is exemplar because it points to the pervasiveness of hyperconnectivity in this kind of lifestyle. So, the interactions *through* and *with* technology are conjuncted and take the shape of a lifestyle in smart living.

But why is this smart living’s technological pervasiveness conducive to loneliness? Focusing on the constant presence of digital connections in Stella’s living environment makes it clear that technological devices like Cyra are not just extended tools through which Stella can communicate with friends. Instead, their constant and recursive employment and their pervasive presence in Stella’s living space foster a needy attitude of constantly searching for supporters to fill her days and nights on Stella’s part.^13^ She does try to counteract this neediness with interactions, but is not satisfied by them.

As evident in this example, extended loneliness does not result from an actual lack of connections. Rather, it is the complex affective experience of both lacking and longing for relationships while being connected to artificial agents and people online. I take extended loneliness to be a complex affective phenomenon. Different components can be investigated, such as the perception of absence, the evaluation of this absence as something bad/to avoid, feelings of anxiety and fear, or the motivation to seek virtual (immediate) social connection to mitigate this absence. In particular, I start from Svendsen’s conceptualisation of loneliness as an unsatisfied need for connection (Svendsen, 2017) and expand on implications for digitally-enabled interaction.^14^ This philosophical account has an important background in the social needs perspective on loneliness that has been developed in psychology and mental health studies (Perlman & Peplau, 1998). Svendsen’s philosophical conceptualisation stresses the relational dimension of loneliness as the background against which loneliness is “an emotional response to the fact that a person’s need for connection to others is not satisfied” (Svendsen, 2017: 14). In this regard, it is important to mention that there are empirical studies that show that lonely people feel even more lonely after using social media (Amichai-Hamburger & Schneider, 2013).

I think that this conceptualisation of loneliness is very helpful for understanding that loneliness need not stem from a lack of connection. Svendsen conceptualisation is a relational conceptualisation of loneliness. This is also useful for distinguishing it from solitude (Svendsen, 2017: 18–21; 91–94; 101–119).^15^ For Svendsen, being alone is not a necessary condition for the subjective experience of loneliness. It is possible to feel lonely while surrounded by many people if these relations do not satisfy one’s need for meaningful engagement.^16^ So, the experience of loneliness is not dependent on the other’s absence, but can arise in the presence of people whose company is considered unfulfilling. So, there is an absence here, but it is an absence of meaningfulness or of other social goods, as argued by Tom Roberts and Joel Krueger (Roberts & Krueger, 2021). It is not the absence of other people in general. Important for my thesis is that the empirical evidence reported by Svendsen shows that the strongest experiences of loneliness occur when surrounded by others (Svendsen, 2017: 21). The reason is that it is in the social life that one can feel that the connections are unsatisfying. Also, empirical evidence shows that loneliness increases when a person has more friends than she would ideally like to have (Russell et al., 2012). Importantly, this is related to my thesis about extended loneliness as a feeling of hyperconnectivity, as I will show in a moment.

People experience loneliness as an unsatisfied need of social connection in the offline social life as well—this is not a new feeling that is inherent to the online life. However, the pervasiveness of hyperconnectivity in smart living augments the extension of loneliness to a lifestyle. My claim is that with the move toward smart living this kind of social loneliness becomes more pervasive and ubiquitous. Also, although online loneliness shares some similarities with the feeling of loneliness that can be experienced in an offline crowd, there are significant differences. There are connections in extended loneliness; it is not a feeling of estrangement from the others we are surrounded by, which is more typical of offline loneliness experienced in crowds, for instance when having no one to speak to at a party. Online loneliness’ overabundance of connections, their sheer numbers, the time they consume, and the different types of media they involve make the subject feel lonely and at the same time seek for new connections. Moreover, it is important to stress that the blend of online and offline life can transform the experience of the loneliness in a crowd as well, since it is quite likely that the subject will attend a party with strangers, for example, having her smartphone at hand to fill any gap in offline conversations with online interactions. In this case, she will not only feel the estrangement from the people at the party, but she also has all her online social networks with her. This could be a support (because she knows, for instance, that if nobody speaks to her, she could always chat with someone online). But it could also exacerbate the feeling of loneliness since it reminds her of the meaningful relationships she longs for but cannot find online or offline.^17^

Moreover, for Svendsen loneliness is an emotion that has a specific phenomenology. In particular, he describes it as a kind of sadness, a painful feeling of discomfort indicating that one’s need for a meaningful relationship is not being met. Accordingly, loneliness is “a perceived lack of closeness to others” (Svendsen, 2017: 17).^18^ Further, I think that focusing on loneliness as a subjective feeling is also important because it shows that it is not just the objective absence of connections (as in solitude), but it is about the *how* of the experience, namely the first-person perspective on the world (Zahavi, 2005, 2014). That is why a relational account of loneliness is not contradictory. Loneliness is in fact a feeling of discomfort when the connections are not fulfilling. But connections might be there. The evaluation of something as disheartening is in fact dependent upon the subject’s concerns and matter of significance.^19^

It might be argued that in the networked life one has many ties and so one cannot feel lonely. I follow Turkle (2011) in my reply to this objection claiming that there is a significant qualitative difference between fully being in a relationship and being connected. Turkle, thinking in particular about teenagers, argues that in online connection teenagers experience a “relationship with less” that they became accustomed to through engagement with speaking toys since their early childhood. This is a merely unilateral and instrumental relationship that does not require two people to meet each other in their wholeness, thereby building a meaningful relationship.^20^ The result is that teenagers are never satisfied with these connections, and they then constantly look for new ones, particularly online, thereby becoming what I call hyperconnected.

At this point, I add an essential qualifier about extended loneliness. The longing for connection as a necessary feature of loneliness is what urges one to search for more fulfilling relationships. However, in extended loneliness, the exaggerated number and omni-pervasiveness of shallow connections exacerbate the feeling of loneliness. So, the longing for connection is a key feature of loneliness but it acquires a specific trait in extended loneliness. It is hyperconnectivity that provides the essential quality to loneliness as extended because, being hyperconnected, the agent realises most clearly how her constant and sometimes exhausting search for connection does not fill her emotional void. Hyperconnectivity is the use of many systems and devices so as to ensure constant connection to social networks and other digital environments. The focus is indeed on the pervasiveness of being always connected in smart living, but also on the exaggerated numbers of connections that, on my analysis, are loneliness-conducive because they do not lead to meaningful relationships.^21^ This also helps in understanding the crucial difference between establishing mere connections and establishing meaningful relationships. A meaningful relationship replies to personal needs and concerns, but it also supports the creation of the good life by fulfilling social goods. So, it is important to stress that the quantitative dimension of hyperconnectivity is grounded in a qualitative dimension, namely the lack and longing of meaningful relationships. Finally, hyperconnectivity makes one feel disconnected from others.

So, extended loneliness is a feeling of hyperconnectivity in the networked life. It is not the feeling that can emerge in social isolation; on the contrary, it is what can only be experienced in overwhelming social connection.^22^ This has implications for a conception of loneliness, for it suggests that the need for relationships can be experienced both in situations in which the other is absent, but also in situations in which too many others are (virtually) present. In the case of extended loneliness, the presence of too many others as virtually present makes it a feeling of hyperconnectivity. As it has been stressed by Svendsen (2017:9) referring to Simmel, loneliness does not imply a lack of community, but rather an unfulfilled ideal of community. Or, referring to Dotson (2017), this also means that what one experiences is a thinning out of community since the thick community of the past has been replaced by networked individualism, a new form of social belonging that is experienced online. For Dotson, this means that togetherness is not a social good anymore but a private responsibility of an individual that makes herself marketable in order to take part in “sporadic meetups with fragmented groups” online (Dotson, 2017: 7).

## Longing for connection

Longing for connection is a fundamental feature of loneliness. Here I need to explain how longing can be extended. To do so, I need to explain the relation between longing and hyperconnectivity. Hyperconnectivity is constitutive of extended loneliness as its vehicle, not just its cause. This means that loneliness is felt through the overabundance of unsatisfying online relationships mediated by the pervasiveness of technological devices in one’s lifestyle. But hyperconnectivity is the result of longing for connection, too, because it is this longing that makes one keep searching for even more connection while being dissatisfied with the ones already possessed. Longing for connection is thus the most fundamental vehicle of extended loneliness since it is what kindles hyperconnectivity. Finally, it is what makes the phenomenal coupling unsatisfactory and distressing. In the case of extended loneliness, the fusion with technology produces a feeling of failed togetherness because the expressive environmental structure one is coupled with—in my case Stella’s smart flat—is conducive to too many swallow relationships. So, when there is hyperconnectivity, at least one key feature of loneliness is extended, namely its volitional component, i.e. longing for connection as a dispositional state.

I cannot develop this active externalist account of longing further here.^23^ For the present aim, it is enough to point to the active dimension of longing that leads to, for example, surfing online, searching for friends and posting beautified pictures on social networks to attract new followers. At the same time, some online platforms boost this longing for connection. I am not simply referring to online dating platforms,^24^ but to any platform that, by being designed as a community platform, triggers the need for more connections because having just a few connections give the user a feeling of being invaluable or lacking.^25^ In this case, the longing for connection would not be an expression of an inner desire for companionship, but a response to the social pressure to accumulate as many connections as possible.

Longing is not simply desire. It implies a strong desire for something difficult to obtain. It is an experience of unfulfillment. So why is a meaningful relationship unattainable online? Why is this online longing unsatisfied?

Fuchs (2014) has argued that in the online environment, there is no real togetherness because one misses primary empathy that is fundamentally grounded in intercorporeality. What is missing is the bodily contact with another person that is crucial for interaffectivity (Fuchs & De Jaegher, 2009).^26^ Fuchs also specifies that human empathy is not bound to immediate intercorporeal contact, but at the same time, he stresses that in this case it can bring some problematic outcomes, as projecting fictional emotions.^27^ Among these worrying outcomes, I claim that there is an increase in loneliness. The reason is that most of the time, enjoying an emphatic encounter is vital for not feeling lonely because one can fulfill one’s needs of recognition. Loneliness is boosted in online sociality because one tries to overcome this lacking empathy by relying upon quantification (creating more connections). But in doing so, one enters a vicious circle: the more one searches for a connection online, the more one feels lonely. This is the core of my argument for a relation between longing for connection and hyperconnectivity: a lack of fulfilling empathic relationships kindles the longing for connection and finally leads to hyperconnectivity.

As I said at the beginning, I do not want to make an absolute and normative claim about the fundamental feeling of online sociality. What I want to stress is that a transition to smart living, such as during the pandemic, can bring new forms of loneliness, namely those arising from an overabundant and sometimes quasi-exclusive online sociality. The result is that immersion in online atmospheres, their pervasiveness in terms of time and space – as in the Stella and Cyra case – foster loneliness. Longing for connection is thus a key feature of the feeling of extension in the case of online sociality and it can take the form of extended loneliness under the circumstances I described.

Having explained in what sense I take loneliness to be extended, namely as an unsatisfactory phenomenal coupling with technology triggered by hyperconnectivity, I use the next section to further analyse its phenomenology. As will become evident, I treat extended loneliness not just as an “emotional episode” of loneliness but as a more pervasive atmospheric feeling that can become chronic, that is an existential feeling of lacking of and longing for relationships while in connection, when hyperconnectivity becomes the mark of a lifestyle.

## Extended loneliness as an existential feeling

By proposing the notion of “existential feeling”, Ratcliffe (2005, 2008) has focused on a range of affective states that are concerned with how one finds oneself in the world. The philosophical root of this notion is the Heideggerian *Befindlichkeit*, a long-lasting affective attunement. This affective attunement orientates the subject’s experience and discloses how the subject relates to the world, to other people and to itself. Crucially, for Ratcliffe, an existential feeling amounts to a felt sense of belonging to the world (Ratcliffe, 2012). When applying this concept of an existential feeling to the case of extended loneliness, it becomes possible to appreciate how pervasive online life is incorporated into the structure of the how we emotionally encounter the world in general. This view also clarifies the specific meaning of extension I am employing here, that is phenomenal coupling through immersion in a specific expressive environmental structure. The vehicle of the extensions are the technological devices, as in the extended cognition’s standard approach. Importantly, however, I do not take them as individual items that a user employs for fulfilling certain tasks; on the contrary, and in agreement with Slaby’s approach to phenomenal coupling (Slaby, 2014), they are the constitutive key features of an expressive environment, in my case the one of a smart flat and, more generally, a smart lifestyle. The vehicle of loneliness hence is not just a technological device but the immersion into a hyperconnected life. So, when hyperconnectivity becomes a lifestyle, for example by being embedded in a living space as in Stella’s case, extended loneliness becomes an existential feeling.

Let us start by asking why extended loneliness is an existential feeling that can be described as in the background. First, an existential feeling is in the background because it is pre-reflective. If one would be fully conscious of it, loneliness would not spread, for, in order to have an extended feeling of loneliness, the agent has to be unaware of it or, put it positively, the device through which this loneliness is created should be phenomenologically transparent, as I have already explained. This sheds light on a fundamental difference to loneliness as a consciously perceived feeling. Most of the time, extended loneliness is not explicitly attended by the agent. It is felt in the background, in a pre-reflective way.^28^

Second, claiming that extended loneliness is a background existential feeling implies that it is not just an emotional episode. This means that the background feeling of loneliness can become part of the self-world experience, regulating and sometimes fixating the how of experience in specific situations or environments.^29^

Being lonely in the background also means that other feelings can be in the foreground. For example, the device that makes one lonely also makes one feels entertained, curious, and relieved. So, there can be a mix of feelings and very often, the more positive ones cover up the negative feelings. Moreover, as Turkle has claimed, users usually love their technological devices (Turkle, 2011). This love makes agents unable to acknowledge possible adverse outcomes that can arise from their use of technology. It also induces the agent to form new habits, transform places and relationships to accommodate her hyperconnectivity—unfortunately, also in an addictive manner, as in the case of the hikikomori.^30^

Sometimes extended loneliness can be felt in the background as a feeling of anxiety, or need for something more, especially in situations of crisis or partial “revelation”, for example when one sees that the virtual assistant’s empathy is just pretence.^31^ But extended loneliness is never fully owned. If that was the case, its power over the subject would be tarnished because one would have the chance to stop what is its main cause: the constant connectedness.

Let us move to the second characterisation. Why is extended loneliness existential? Extended loneliness reveals something about the self by being world-disclosive (Slaby & Stephan, 2008). This means that extended loneliness discloses hyperconnected worlds in which there is a risk of losing the grip on people and things. The self that relates to these worlds thus would be a self that cannot find meaningful relationships and whose existence would be impoverished because one’s action possibilities are reduced. In this regard, Ratcliffe has claimed that an existential feeling is an experience of worldly possibilities (Ratcliffe 2011). The online worlds present infinite possibilities and so one may hope to find there the meaning one is looking for. But, as I hopefully clarified in this paper, it can happen that this continuous longing for connection becomes a source of loneliness. An existential feeling is a way of belonging to the world. But in the case of extended loneliness, a failure of belonging is experienced instead. Still, I claim that extended loneliness is an existential feeling, but in an inverse way. Not as a luminous feeling of belonging to the world, but as the gloomy feeling of not being seen by anybody.^32^

These features help in defining the meaning of extension I employ here. The phenomenal coupling with technology as immersion in the online atmosphere of smart living is then experienced as a background existential feeling. This means that, in the case of extended loneliness, hyperconnectivity, that I have claimed to be the fundamental vehicle of extension, assumes an existential relevance. That is as a failure of worldly possibilities in terms of fulfilment of social needs. Speaking about the tethered self, as Turkle does, makes more sense now that we have this conceptual framework. Extended loneliness is not just an episode of feeling lonely, but it is the way in which a tethered self finds itself in the online worlds. The phenomenal coupling is thus constitutive of the how of the experience of the tethered self and in so doing hyperconnectivity can be the fundamental unsatisfactory way of connecting to others.

## Conclusion

In this paper, I shed light on the phenomenon of extended loneliness that has become especially prevalent during the Covid-19 pandemic. I argued that this type of loneliness experienced by the tethered self is extended because it is constituted by the overabundance of digitally-mediated interactions of the networked life in a smart living lifestyle. It is a specific kind of loneliness that is felt while in connection with others. In more technical terms, it is extended because it is mediated through technological devices which have become phenomenologically transparent and are thus automatically trusted, used, and become crucial components of the user’s lifestyle. I have argued that what is extended is in particular the volitional dimension as a longing for connection that triggers hyperconnectivity. I have explored the feeling of extension as phenomenal coupling and claimed that extended loneliness is a particular feeling of being immersed in the online worlds by inhabiting smart spaces. Exploring its phenomenology, I described it not as an episodic feeling but a background sense of lacking and longing for relationships while being in connection. Extended loneliness is hence an existential feeling.

As I showed, extended loneliness is not a necessary component of the networked life, but it becomes especially relevant for hyperconnected users. The result is that technologies designed to foster relationships can unfortunately become technologies of loneliness.^33^

But this should not be the last word. For example, there is the possibility that the pandemic, exposing users to phenomena like “cyber indigestion” or “Zoom fatigue”, can kindle the desire to build meaningful relationships, both online and offline. This means that there is the possibility that once the excessive number of online connections becomes even more obvious and ubiquitous, users might become aware of the problem and find the strength to look for alternative ways of forming relationships. Awareness of cyber-indigestion can make extended loneliness emerge from the background and let the user see it. Gaining such awareness lies not just in the power of the individual. A collective effort and social responsibility in transforming detrimental (online) cultures of relating are necessary. My contention is that this conceptual and phenomenological analysis of extended loneliness can be the starting point for ameliorative projects regarding the ethics of the tethered self.

## Footnotes

1 *The Guardian*, 21st March 2021, interview by Ian Tacker.

2 Although simulation theory is mainstream in designing expressive anthropomorphic robots, there are new research projects that are employing different frameworks for modelling robots’ social skills and emotions. For example autopoiesis (Man & Damasio, 2019) and participatory cultural robotics (Ornelas et al., 2022, Candiotto & Mansouri, 2022 forthcoming) can better meet the need of building thick relationships between humans and social robots.

3 See Dreyfus (2009) for an example of a strong emphasis on online-offline differences. See Eklund (2015) for the opposite claim about the constitutive blend of online-offline.

4 For a comprehensive discussion of the debate, see Colombo et al., (2019).

5 Memory processes are the key-cases discussed by the critics and proponents of the extended mind model, also for testing the hypothesis of socially extended processes in transactive memory. See for example Sutton et al., (2008). Regarding memory processes and the extended self, see Heersmink (2017; 2018) and Wilson & Lenart (2015).

6 This thesis should not be confused with that of the conscious extended mind according to which consciousness extends through the body and into the world. Although it has been advanced by some scholars (for instance, Ward 2012), especially in enactive frameworks, the conscious extended mind view has been explicitly challenged by Clark (2009) for whom automatic endorsement remains a core tenet of the extended mind hypothesis.

7 On the different current conceptualisations of extended emotions see Candiotto (2016) and Krueger & Szanto (2016).

8 Along a similar line, but within the pragmatist tradition, see Candiotto & Dreon (2021).

9 It is important to stress that, in line with the socially extended mind framework (Gallagher, 2013), for Slaby (2014) the extension is not reduced to technological devices, but it also happens with other people and institutions. I do not discuss the socially extended mind approach here, although it might be useful to study extended loneliness along this line in the future. The reason is that the orthodox meaning of extension through technological devices is already so much underexplored in terms of the feeling of extension that I think we need to start from it.

10 Snell (2015, 2017) argues that the most significant explanatory variable in the history of loneliness is living alone.

11 This is historically determined by our networked society, but we can also find its root in the need for belonging that pertains to the human species. On the evolutionary basis of loneliness as a primal experience of needing to belong in tribes, see Cacioppo et al., (2009); Cacioppo & Patrick (2009). In clinical studies and therapy, emotional loneliness is traditionally related to attachment theory and seen as resulting from a lack of deep, nurturing relationships with other people (Weiss, 1974). See also Ratcliffe (2016) for a philosophical discussion of how loneliness arises from our intrinsic sociality and a criticism of loneliness as dependent upon an isolated self-awareness as defended by Mijuskovic (2015).

12 It is for me also important to stress the interaction *with* AI and not *through* AI because this is what is going on now. The interaction *through* AI is already here since 10 years at least.

13 Stella also has a chatbot that replicates her dead grandmother’s conversational style and tone with whom she interacts before sleeping as part of her evening ritual.

14 I thus take extended loneliness as historically and culturally embedded. For an analysis of loneliness that primarily considers its historical configuration, see Alberti (2018).

15 On the differences between loneliness and solitude, see also Alberti (2018).

16 This philosophical conceptualisation is in line with the belongingness hypothesis for which humans have almost universal need to form and maintain at least some degree of interpersonal relationships with other humans and the understanding of loneliness as social pain and in evolutionary science. See Baumeister & Leary (1995); Cacioppo et al. (2009); Cacioppo & Patrick (2009).

17 Many sociological studies about crowd loneliness have been developed since the 50s within the reflections about the societal transformations in modernity. See Riesman et al., (2001).

18 Here Svendsen is referring to the cognitive discrepancy model for which loneliness occurs when individuals perceive a difference between their desired and actual levels of social involvement. See Russell et al. (2012).

19 For emotions as subjective evaluations, see Döring (2010).

20 I do not understand “wholeness” in terms of embodiment,as per Dreyfus (2009), but in terms of existential meaningfulness. An excellent analysis of this comes again from Turkle: “We enjoy continual connection but rarely have each other’s full attention. We can have instant audiences but flatten out what we say to each other in new reductive genres of abbreviation. […] We have many new encounters but may come to experience them as tentative, to be put “on hold” if better ones come along. Indeed, new encounters need not be better to get our attention. We are wired to respond positively to their simply being new.” (Turkle, 2011: 279–280).

21 On the empirical research about the impact of hyperconnectivity on the brain, and in particular on the increase of loneliness provoked by the excessive, yet passive use of technology, see Moretta & Buodo (2020). Interestingly, this empirical research shows that a moderate use of technology, especially by users who engage with others, rather than just passively consume content, can increase social connection.

22 Of course it can happen that one is actually alone in the social media, for example by having only four followers in Instagram. This simply shows that one can feel lonely when he is alone online, but it is not the case that one feels lonely only if she is alone. On the contrary, and this is the phenomenon I am describing, one can feel lonely when she is surrounded by too many unsatisfactory relationships.

23 Although there is not much about this third dimension of extension, beyond the cognitive and the affective, in the extended mind research program, some externalist accounts of motivation can help in this regard. See Battaly (2018), Candioto (2019).

24 See Ben-Ze’ev (2004).

25 Some episodes of the sci-fi series “Black Mirror” are quite representative of it.

26 Notably, by embodiment Dreyfus (2009: 69–74) means all aspects of our finitude and vulnerability. He claims that we miss this kind of embodiment online. I think that there is indeed a risk of concealing our vulnerabilities online, for example by employing beautifying tools and writing spectacular posts for social media platforms in which one provides an empowered image of oneself. Still, I take extended loneliness to be an embodied and lived experience that has its own phenomenology. Therefore, I do not subscribe to the non-embodiment thesis of online life. Instead, I suggest exploring its peculiar phenomenology and embodiment. On the embodied dimension of online experience, see also Osler (2020).

27 This is the case of what Fuchs calls “extended empathy”, namely the empathy that is based on the imaginative representation of the other. See Fuchs (2014).

28 On the varieties of pre-reflective awareness in emotion experience, see Colombetti (2011).

29 This is related to chronic loneliness. Analysing chronic extended loneliness is beyond the scope of this paper and would require a deep investigation into its psychopathology. See Svendsen (2017) for a preliminary overview of chronic loneliness.

30 Hikikomori is a severe form of social withdrawal. This phenomenon has been frequently described in Japan where increasingly many teenagers and young adults refuse to go outside their rooms. The risk of hikikomori has been studied in relation to Internet addiction by Tateno et al. (2019).

31 This crisis can trigger processes of habit-revision and habit-transformation. See on this Candiotto & Dreon 2021. See also Stephan (2015) regarding artificial empathy.

32 Ratcliffe conceptualisation of existential feelings has also prompted the study of schizophrenia and depression as altered existential feelings. See Ratcliffe (2011).

33 It could be argued that some of these devices have been designed exactly to attain compulsive behaviours, but I cannot investigate this possibility further here.
